# Professional Notes

**Published:** 1887-10-01

**Authors:** 


### Professional Notes.

By a Physician.

Arsenic is now very generally used in practice. Mr. Jonathan Hutchinson mentions an interesting fact in this connection. It is well known that in cases of arsenical poisoning curious forms of paralysis may follow; the regions affected are often strictly limited, and are frequently confined to one side only. It is an equally familiar fact that herpes is a nerve-affection, and that it is often brought out by arsenic. Most herpetic affections occur but once in a lifetime at the same place. Herpes labialis, however, is of frequent recurrence, and Mr. Hutchinson asserts that he does not know anything so efficacious in preventing this recurring tendency as arsenic. Arsenic, therefore, will both cause and cure herpes. Is this a fact which favours the *similia similibus* theory?

Ringworm is said by Dr. Hutchinson, of Anderson's College Dispensary, Glasgow, to have been promptly cured by the application of Tincture of Siegesbeckia Orientalis. The tincture is mixed with its own volume of glycerine, and rubbed energetically into the affected part several times a day. In the experiments hitherto tried epilation was not found necessary.

Dr. C. R. Illingworth, an enthusiastic therapeutist, speaks favourably of the use of the biniodide of mercury for certain scarlatinal throats. To two ounces of the solution of bichloride of mercury he adds carefully, drop by drop, a few drops of a 1 to 4 solution of iodide of potassium or sodium, shaking the liquid after the addition of each drop. On precipitation a cloudy red liquid is obtained, and to this he adds half an ounce of glycerine to keep the particles of biniodide in suspension. The mixture is applied twice a day, with the result of arresting the inflammatory and ulcerative processes in a very short time. He is of opinion that the same application would prove of great service in diphtheria, which is very probable.

Strophanthus is coming into extensive use as a substitute for digitalis. Professor Fraser continues to believe in its value, and his views are confirmed by many other observers. In dilated conditions of the heart and the dropsy resulting therefrom it seems often to be of great service, restoring the regularity and steadiness of the cardiac movements more promptly than digitalis. In anginal pains it is said to give relief. Few ill-effects have been recorded from its administration, though it has been known to produce nausea, and vomiting to such a degree as to be entirely unavailable for therapeutic purposes. It may be given as tincture with water alone, or combined with other medicaments which seem to be indicated at the moment. A very easy way of experimenting with the drug is the administration, two or three times a day, of one of Burroughs and Wellcome's tablets.

Dr. Austin Flint, of New York, speaking at the International Medical Congress on the subject of fever, expressed the decided opinion that fevers are due to a certain specific cause, probably micro-organisms. He considers that at present we have no means of destroying these, and that the aim of treatment must be twofold, viz. (*a*) to moderate their action, and (*b*) to sustain the vital powers. There is nothing new in these pronouncements, for the best of all reasons that true science at present knows of nothing new on the subject. The ataxic symptoms in fever are proportionate to the elevation of temperature. These must be ameliorated by treatment chiefly directed to reduction of temperature, as by the abstraction of heat by cold or the use of antipyretics. During the period of inanition the consumption of the tissues in the production of animal heat is in a great measure saved by the increased production and excretion of water. Alimentation is difficult, mainly on account of the derangement of the digestive organs. Food easy of digestion, or partially digested, is therefore imperatively demanded. Dr. Flint considers that not only is alcohol promptly oxidised but it is capable of absorption without digestion. So far as it is oxidised in the body it supplies matter consumed in the production of heat, and saves tissue destruction. Here again is an idea by no means new, that alcohol is an antitryptic, or preventer of tissue waste. Oxidative alcohol involves the formation of water, and thus limiting tissue destruction, tends to restore heat-production processes to the normal. This view of alcohol in the treatment of fever is endorsed by the experience of very many capable observers.

Dislocations are often very difficult of reduction, and the operation frequently causes agonising pain. Dr. Geo. C. Kingsbury has used cucaine, subcutaneously injected, with results which gave him decided satisfaction. In one case a mason dislocated his elbow-joint by a fall from a scaffold. The skin was made so tense that the bones seemed as if they must protrude. The pain was intense, and the patient could not tolerate the least manipulation. Five minims of an 8 per cent. solution, injected deeply near the head of the radius, produced complete local anæsthesia, and at the same time relaxed the marked muscular tension. The dislocation was then promptly and quite painlessly reduced. In the case of a lad who had a fish-hook deep in his finger, a few drops of the solution injected near the spot destroyed the sensibility, so that the hook was easily extracted without the boy feeling the operation at all. It is clear that in cucaine the profession has a substance which may, without the risks attendant upon the administration of chloroform, save a vast amount of suffering, and bring much credit to the practitioner.

According to Dr. Langdon Down, with whom Dr. Fletcher Beach coincides, habitual intoxication in a parent is a common cause of idiocy in children. It is stated on authority that on the removal of the spirit duty in Norway insanity increased 50 per cent., and idiocy 150. Probably these figures cannot be absolutely relied upon as having been scientifically verified. But there is abundant evidence to show that general propositions affirming a constant relation between parental drunkenness and idiocy in the next or succeeding generations are indisputable. Dr. Langdon Down, as the result of a prolonged series of observations, is convinced of the baneful influence of intoxication in this direction.

Acne is one of those disorders which no patient will endure if he can help it. After trying his own medical attendant for a few weeks he is sure to go elsewhere if no improvement takes place. Iodoform mixed with simple ointment will sometimes cure obstinate cases, if well rubbed in from time to time. The suggestion, however, is not of great practical value, because few patients can be persuaded to carry about with them the unbearable odour of the ointment. Small repeated doses of belladonna are said to cure acne of the face. Ergot, too, is believed in by some; and both these drugs it is thought correct the local vasomotor irregularity. Dr. Lauder Brunton considers bromide of potassium useful when given in moderate doses. A not unnatural curiosity might inquire whether an induced attack of acne is not conversely worth trying as a cure for bromide eruptions.

